# Bullying Prevalence among Secondary School Children in Khamis Mushait City, Southwestern Saudi Arabia

**DOI:** 10.3390/bs11100134

**Published:** 2021-10-01

**Authors:** Mohammed Abadi Alsaleem, Huda Aied Alhashem, Safar Abadi Alsaleem, Ahmed A. Mahfouz

**Affiliations:** 1 Department of Family and Community Medicine, College of Medicine, King Khalid University, Abha 61421, Saudi Arabia; mabade@kku.edu.sa (M.A.A.); asalslim@kku.edu.sa (S.A.A.); 2 Family Medicine Department, Aseer General Directorate of Health Affairs, Abha 62523, Saudi Arabia; Huda-99Hh@hotmail.com

**Keywords:** bullying, adolescents, Saudi Arabia

## Abstract

Bullying is a type of behavior that involves frequent, hostile activities expected to harm another person physically, mentally, or emotionally. Bullying behavior uses force, pressure, or threats to maltreat, forcefully dominate, or terrify another individual. The aim of this study was to assess the bullying prevalence and related features among secondary school pupils in Khamis Mushait city, southwestern Saudi Arabia. A cross-sectional investigation was performed among governmental and private secondary schools in Khamis Mushait city. Data were gathered from the study pupils using a pre-structured questionnaire. Bullying was assessed using the school climate bullying survey. The study included 300 secondary school students (163 females and 137 males). The overall rate of bullying was 64.7% (95% CI 59.1–69.9). The most prevalent type of bullying was verbal (41.7%, 95% CI 36.0–47.5) followed by physical (17.0%, 95% CI 12.9–21.7), and social (6.0%, 95% CI 3.6–9.3). Males had more than two times greater probability of having been bullied than females (aOR = 2.522, 95% CI 1.408–4.518). Similarly, students in first-level grade classes had more than three times greater probability of being bullied victims than those in the higher classes, i.e., second- and third-level grade classes (aOR = 3.417, 95% CI 1.159–10.07). More than half of the students tell teachers when other students are being bullied (53.7%) and tell a teacher or staff member at the school if they are being harassed (53.6%); teachers are doing anything they can to help if they are told that a student is being bothered (58.7%), and teachers are making clear to students that bullying is not tolerated (52.3%). In conclusion, in the present study, we reported a high prevalence rate of bullying among secondary school pupils in Khamis Mushait city, southwestern Saudi Arabia. Concerted efforts among teachers and health care providers in the region should be mandatory to deal with the problem.

## 1. Introduction

Bullying behavior is frequent, hostile activities expected to harm another person physically, mentally, or emotionally. Bullying behavior uses force, pressure, or threats to maltreat, forcefully dominate, or terrify another individual [1]. Another simple definition of bullying was given by “Dan Olweus”. He stated “A student is being bullied or victimized when he or she is exposed, repeatedly and over time, to negative actions on the part of one or more other students.” It is a negative action when someone intentionally inflicts, or attempts to inflict, injury or discomfort upon another—basically what is implied in the definition of aggressive behavior [2]. The behavior is often recurring and persistent. One fundamental requirement is the awareness of a discrepancy of physical or social supremacy [3]. This disparity differentiates bullying from non-bullying behavior [4]. Bullying is a subgroup of violent behaviors described by three main criteria: aggressive intention, the discrepancy of influence, and recurrence over time [5]. Bullying can take different approaches ranging from one-on-one, personal bullying to band bullying, called mobbing. A bully may have one or more assistants who want to support the chief bully in their bullying actions. Bullying in schools and places of work is also referred to as “peer abuse” [6]. Bullying has been defined in many different ways. Bullies’ intentions to change their behaviors following a discussion with a school personnel member are higher when they perceive that the adult conducting the intervention attempted to raise their empathy for the victim and condemned their behavior [5]. In the United Kingdom, there is no legal description of bullying, while some states in the United States have laws against it [7]. In Saudi Arabia, there is no legal description of bullying. Bullying is divided into four essential types: abuse-psychological (sometimes called emotional or interpersonal), vocal, corporeal, and virtual (cyber) [8].

Bullying has been studied worldwide. A study by [9] assessed the magnitude of bullying and victimization in 40 countries worldwide among students aged 11 to 15 years. The study revealed that bullying was a worldwide problem, with the occurrence ranging from approximately 9 to 45% among males and 5 to 36% among females. Other studies in less developed countries have revealed a similar conclusion [10,11]. In developed countries, studies have shown varying figures. In Italy, a study showed a prevalence of victimization of 37%, whereas that for perpetration was 21% [12]. In a meta-analysis on bullying and cyberbullying prevalence across contexts in Western countries with an overall sample of 335,519 youth (12–18 years), the authors estimated a mean prevalence of 35% for traditional bullying [13]. In Saudi Arabia, a study reported, on the one hand, that most students were participating in bullying and victimization (51.5%), while 41% of students were merely victims, and only1.5% of students were intimidators. On the other hand, 6% of students were not involved in bullying or victimization [14].

Bullying among secondary school students has been recorded and investigated by many researchers. The method of bullying depends mainly on gender, as boys tend to use direct forms of peer annoyance, such as beating, punching, or kicking. At the same time, girls are more likely to use hidden or indirect forms of harassment as the strategy in friendships or relationships with peers, such as initiating rumors or malicious gossip [15,16]. Adolescent victims of bullying are more predisposed to mental health problems, including suicidal ideation and behavior, which can continue later on during their life [17]. The victims also may have other destructive behaviors with adverse health effects, such as substance use [18,19].

Secondary school students are representative of the late adolescent period. Data regarding bullying among adolescents in the Aseer region are scarce and even lacking. For many cultural reasons, this population data are crucial and may offer new insights into the broader bullying described in the literature. Studying bullying behaviors could significantly minimize children’s and adolescents’ mental health problems and could also prevent psychiatric and socioeconomic difficulties in adulthood. In the present study, we aim to assess bullying and related factors among secondary school students in Khamis Mushait city, southwestern Saudi Arabia. The selected population is representative of adolescents in different regions in Saudi Arabia. The study should pave the way to understanding of bullying among similar populations in Saudi Arabia.

## 2. Materials and Methods

### 2.1. Study Design and Setting

A cross-sectional study was performed among governmental and private secondary schools in Khamis Mushait city.

### 2.2. Target Population and Sampling

The study targeted all secondary school students (male and female) in Khamis Mushait city. With an anticipated proportion of 50% and absolute precision of 6% at 95% confidence, the sample size desired for the survey was calculated to be 267 students [20]. The response level is the expected occurrence of the outcome or event of concern. Data regarding expected prevalence rates should usually be taken from the literature. When this information is not accurately obtainable, the figure that increases sample size is used, which is 50% [21]. A sample size of 300 students was planned for the study to account for potential nonresponse. A three-stage stratified random sampling technique was applied. Stratification depended on school nature (private and governmental) and student gender (male or female). Three districts (out of five educational districts) were randomly selected in the first stage of cluster sampling. For the second stage of cluster sampling, four schools were randomly selected in each district (three governmental and one private), which was based on the total numbers of governmental schools (92) and private schools (33) (3:1). For the third stage of cluster sampling, two grade classes were randomly chosen within each school (out of a maximum of five grade classes), and all students were requested to participate in the survey. The average number of students per class ranged from 10 to 14 students.

### 2.3. Study Tool

The data were collected from the study students using a pre-structured questionnaire. The questionnaire covered students’ sociodemographic data, including age, gender, grade, type of school, parent education, parent job, and family monthly income. Bullying was assessed using “The School Climate Bullying Survey (SCBS)” [19]. The items included: frequency of bullying or being bullied by others, whom the student told about being bullied, locations where bullying occurs, school climate items, willingness to seek help, and the prevalence of teasing and bullying. The scale was translated into Arabic by a group of experts. The scale was translated and back translated. A pilot study including 50 students was conducted to confirm the validity of tools, test for reliability, and to assess the tool’s clarity and the time to complete questionnaire. The calculated Cronbach’s alpha was 0.83 and the inter-item correlation was 0.71.

### 2.4. Data Analysis

Data were studied using the SPSS software package, version 22 (IBM Corp. Released 121 2013. IBM SPSS Statistics for Windows, Version 22.0. Armonk, NY: IBM Corp.). Frequencies, percentages, and accompanying 95% confidence intervals were used. Crude odds ratios (cOR) and accompanying 95% confidence intervals were calculated. Similarly, adjusted odds ratios (aOR) and accompanying 95% confidence intervals were computed (using multivariable analysis).

## 3. Results

### 3.1. Description of the Study Sample

The present study included 300 secondary school students. They included 163 females (54.3%) and 137 (45.7%) males. Their age ranged from 15 to 18 years. More than half of them (163, 54.3%) had from one to five siblings and had a birth order of more than two (165, 55%). The most frequent father’s education level was secondary (103, 34.3%). More than half of them were governmental employees (172, 57.3%). The most frequent mother’s education level was less than secondary (121, 40.3%). More than half of their mothers (163, 54.3%) were not working (just housewives). The most regular monthly income (117, 39%) was from five thousand to ten thousand SR.

### 3.2. Prevalence of Bullying

The present study showed that the overall bullying prevalence rate was 64.7% (95% CI 59.1–69.9). Table 1 shows that the most prevalent type of bullying was verbal (41.7%, 95% CI 36.0–47.5) followed by physical (17.0%, 95% CI 12.9–21.7) and social (6.0%, 95% CI 3.6–9.3).

### 3.3. Bullying Correlates

Table 2 shows the univariate and multivariate analysis of sociodemographic bullying correlates. The analysis showed that after adjusting for other sociodemographic factors in the multivariable logistic binary analysis, males had more than two times greater probability of having been bullied as compared with females (aOR = 2.522, 95% CI 1.408–4.518), which was significant. Similarly, students in first-level grade classes had more than three times greater probability of being bullied victims than those in higher classes, i.e., second- and third-level grade classes (aOR = 3.417, 95% CI 1.159–10.07).

### 3.4. Bullying Environment

Table 3 shows the bullying environment among the study sample. The study showed that more than two-thirds of students believed that “bullying is a problem in the school” (256, 88.3%), “students are exposed to a lot of harassment about different topics in the school” (240, 80.0%), “students are often harassed because of their race or tribe in the school” (231, 77.0%) and “students at school often get upset about their clothes or their physical appearance” (228, 76.0%).

Regarding behaviors towards bullying, the table shows that more than half of the surveyed students stated that “they are telling teachers when other students are being bullied” (161, 53.7%), “they are telling a teacher or staff member at the school if being harassed” (160, 53.6%), “teacher doing anything to help if being told that I have being bothered” (176, 58.7%), “presence of adults in the school to turn to in case of any personal problem” (154, 54.3%), and “teachers are making clear to students that bullying is not tolerated” (157, 52.3%).

Concerning attitudes towards bullying, the table shows that minority believed that “students who are bullied often deserve it” (87, 29.0%), “bullying is sometimes fun to do” (61, 20.3%), and “it is feeling good to hurt someone” (55, 18.3%).

## 4. Discussion

The present study showed that the overall bullying rate among secondary school children in southwestern Saudi Arabia amounted to 64.7%, and the most prevalent type of bullying was verbal (41.7%), followed by physical (17.0%) and social (6.0%). The study showed that males had more than two times greater probability of bullying as compared with females. Similarly, students in first-level grade classes had more than three times greater likelihood of being bullied victims than those students in higher classes; second- and third-level grade classes.

In Oman, a cross-sectional report assessed the magnitude of bullying among school pupils in Muscat [22]. The study revealed higher figures as compared with the present study. It was found that 76% of students had experienced bullying one form or another. In nearly one-half of the occasions, the bullying was started by a student of a similar age or older than the victim. The most common type of bullying encountered was verbal (47.7%), followed by misuse (45.9%), physical (43.9%), and, finally, social isolation/exclusion (22.5%). In Brazil, a study [23] assessed the prevalence of bullying among school children. The researchers found a bullying prevalence of 21.26%. A survey on Arab American adolescents in the USA found a bullying prevalence of 30% [24].

A study was conducted among private school children in Riyadh in Saudi Arabia [14] found a bullying prevalence of 41%. In Jeeluna’s study [25], in Saudi Arabia, a nationwide study addressing the health needs of adolescents, 26% of adolescents reported exposure to bullying. In Jeddah, a study [26] reported a bullying prevalence of 41.1% among school children. Cultural differences can explain the observed high bullying prevalence in the present study.

A recent study [27] evaluated data from a worldwide school-based student health survey of school pupils aged 12–17 years, between 2003 and 2015, in 83 countries in the six world health organization regions. The study found a pooled global bullying prevalence of 30.5%. The highest bullying prevalence was observed in the Eastern Mediterranean (45.1%) and African (43.5%) regions. Similar to our results, the study found that bullying was associated with male gender (OR: 1.21) and younger class levels (OR 2.11). Another study, including 40 Western countries, found a bullying prevalence of 12.1%. Similarly, the study found that boys were found higher in being bullied victims [9]. The same findings of boys greater in being bullied victims were reported in the Riyadh study [14]. These results point out the similarity of our findings to other studies in the region or in Saudi Arabia.

The present study found that more students in the first-level grade classes were bullied victims. Another study reported similar findings on data taken from the 2005–2006 Health Behavior in School-Aged Survey in the USA [28]. This phenomenon can be explained by the fact that bullying is an acquired behavior; therefore, students gain and perform different bullying behavior types as they grow older, and therefore the more students are engaged in bullying at younger ages, the more they learn and develop their ways of bullying by starting to use more than one type of bullying.

The present study showed that more than half of the surveyed students mentioned that they tell teachers when other students are being bullied, they tell a teacher or staff member at the school if they are being harassed, teachers are doing anything they can to help if being told that a student is being bothered, and teachers are making it clear to students that bullying is not tolerated.

Teachers are in a persuasive position as educators and agents of socialization, since they assist to foster healthy relationships among students and to prevent unfavorable relations. Teachers are often nearby when an event of bullying happens, and frequently, they are the first adult advocates that students approach [29]. Teachers can respond with several approaches after a bullying occasion, including interfering, examining the situation, not intervening, ignoring, and trivializing the bullying [30]. They can observe bullying incidents, interfere by backing the victim or the bully, and discuss the significance of a positive class environment with the group. Students assume that teachers actively interfere when bullying occurs [12]. The achievement of teacher interference has important insinuations in terms of how students should be effectively supported and how their self-confidence and sense of safety might increase. Trying to expand factors forecasting a successful reaction of teachers to bullying is a main concern to define the most significant constituents for teachers’ preparations [31].

A recent study suggested the following guidelines to maximize teachers’ roles in preventing bullying [32]. Increased understanding of this fact is the first step, along with endorsing awareness about bullying and victimization. Second, increasing abilities and capabilities regarding the efficient ways to interfere after a bullying event seems to be critical. In addition, supporting teacher involvement and observing the process may be relevant to acquire a true consciousness of self-efficacy in teachers’ handlings of bullying and victimization. Third, job satisfaction would result in a significant variable for the daily work in class. Job satisfaction is related to a teacher’s self-efficacy and their sense of observed capability, and both are a primary source for inherent motive and satisfaction.

Health care providers also need to keep an opportunistic eye open for bullying and other forms of aggression when providing general care to their school-aged children. Prevention, identification, and management of bullying and peer violence are essential responsibilities for health care providers. Any contact with a young patient should be viewed as an opportunity to address some of these potentially missed issues.

## 5. Limitations, Future Implications, and Clinical Suggestion

The current study provides useful evidence of the magnitude of bullying in the region. The study population is representative of adolescents in one region. Results will pave the way to understanding of bullying among similar populations in Saudi Arabia. However, there are some limitations that can be addressed in future research. First, the present study employed a cross-sectional design; therefore, future longitudinal studies should be added to observe variation of bullying and correlates among families. Second, our findings are based on a sample in a localized geographical area, and for this reason, the results cannot be generalized all over Saudi Arabia. Another limitation is the use of a single item question for the prevalence of bullying could overestimate the phenomenon. Future research could involve different regions of Saudi Arabia. Moreover, the present study is based on youth assessments with the exclusion of fathers and mothers, a fact that constitutes the major limitation of the present study. Other qualitative data would be useful to better understand family and psychological variables. This study includes only self-report measures that expose results to limitation due to social desirability. Despite these limitations, a number of clinical implications from this study could be suggested that could improve clinical practice and guide future research.

## 6. Conclusions

The present study reported a high prevalence rate of bullying among secondary school pupils in Khamis Mushait city, southwestern Saudi Arabia. Concerted efforts among teachers and health care providers in the region should be mandatory to control the problem.

## Figures and Tables

**Table 1:** Prevalence of bullying and concomitant 95% confidence intervals among secondary school students in Khamis Mushait city, southwestern Saudi Arabia (*n* = 300).

Bullying	Number	Prevalence Percent	95% Confidence Interval
Overall bullying	194	64.7%	59.1–69.9
Verbal bullying	125	41.7%	36.0–47.5
Physical bullying	51	17.0%	12.9–21.7
Social bullying	18	6.0%	3.6–9.3

**Table 2:** Univariate and multivariable analysis of sociodemographic factors associated with bullying among secondary school students in Khamis Mushait city, southwestern Saudi Arabia (*n* = 300).

Sociodemographic Variable	cOR (95% CI)	aOR (95% CI)
Age: 15–16 years vs. more than 16 years	1.464 (0.807–2.556)	0.492 (0.200–1.212)
Sex: Males vs. females	4.115 (2.432–6.963)	2.522 (1.408–4.518) *
Class: first-level grade class vs. second- and third-level gradee classs	2.984 (1.437–6.395)	3.417 (1.159–10.07) *
Number of siblings: 1–5 vs. more than 5	0921 (0.572–1.482)	0.782 (0426–1.433)
Birth order: 1–2 vs. more than 2	1.403 (0.863–2.269)	1.641 (0.869–3.099)
Father education: Less than secondary vs. secondary and higher	2.669 (1.485–2.908)	1.955 (0.890–4.294)
Father occupation: Governmental vs. retired and private	1.419 (0.489–1.928)	2.675 (1.507–4.747)
Mother education: Less than secondary vs. secondary and higher	1.953 (0.748–2.088)	1.331 (0.987–2.001)
Mother occupation: Notworking vs. working	2.105 (1.301–3.405)	1.209 (0.636–2.298)
Monthly income: Less than 10 thousand vs. 10 thousand and more	2.201 (1.347–3.596)	0.805 (0.400–1.619)

cOR, crude odds ratio; aOR, adjusted odds ratio; 95% CI. 95% confidence interval, * significant.

**Table 3:** Bullying environment (perception, behaviors, and attitudes) among secondary school students in Khamis Mushait city, southwestern Saudi Arabia (*n* = 300).

Bullying Environment (Perception, Behaviors and Attitudes)	Number	Percent
**Perception of Bullying**		
Bullying is a problem in the school	265	88.3
Students at school are often get upset about their clothes or their physical appearance	228	76.0
Students are exposed to a lot of harassment about different topics in the school	240	80.0
Students are often harassed because of their race or tribe in the school	231	77.0
**Behaviors towards bullying**		
Students are telling teachers when other students are being bullied	161	53.7
Telling a teacher or staff member at the school if being harassed	160	53.6
Teacher doing anything to help if being told that I have being bothered	176	58.7
Presence of adults in the school to turn to in case of any personal problem	154	54.3
Teachers are making clear to students that bullying is not tolerated	157	52.3
Presence of warning to students who are bullies to stop bullying in case of bullying	158	52.7
Teachers in the school are honestly interested in students	169	59.3
**Attitudes towards bullying**		
Students who are bullied or bullied often deserve it	87	29.0
It is feeling good to hurt someone	55	18.3
It is OK to hit a person who threatens me	184	61.3
Bullying is sometimes fun to do	61	20.3
Whenever I am aggressive, everyone will fear me	161	53.7
I will not have many friends if I am afraid to get into trouble and hostilities	141	74.0

## Data Availability

Data supporting reported results can be requested from the corresponding author upon request.
